# Early Deletion and Late Positive Selection of T-Cells Expressing a Male-Specific Receptor in T-Cell Receptor Transgenic Mice

**DOI:** 10.1155/1990/18208

**Published:** 1990

**Authors:** Hung Sia Teh, Hiroyuki Kishi, Bernadette Scott, Peter Borgulya, Harald Von Boehmer, Pawel Kisielow

**Affiliations:** 1 Department of Microbiology University of British Columbia Vancouver Canada; 2 The Basel Institute for Immunology Basel Switzerland; 3 The Institute for Immunology and Experimental Therapy Polish Academy of Sciences Wroctaw Poland

## Abstract

The ontogeny of T cells in T-cell receptor (TCR) transgenic mice, which express a transgenic αβ heterodimer, specific for the male (H-Y) antigen in association with H-2D^b^, was determined. The transgenic α chain was expressed on about 10% of the fetal thymocytes on day 14 of gestation. About 50% of day-15 fetal thymocytes expressed both α and β transchains and virtually all fetal thymocytes expressed the transgenicαβ heterodimer by day 17. The early expression of the transgenic TCR on CD4^-^8^-^ thymocytes prevented the development of γδ cells, and led to accelerated growth of thymocytes and an earlier expression of CD4 and CD8 molecules. Up to day 17, no significant differences in T-cell development could be detected between female and male thymuses. By day 18 of gestation, the male transgenic thymus contained more CD4^-^8^-^ thymocytes than the female transgenic thymus. The preponderance of CD4^-^8^-^ thymocytes in the male transgenic thymus increased until birth and was a consequence of the deletion of the CD4^+^8^+^ thymocytes and their CD4^-^8^+^ precursors. By the time of birth, the male transgenic thymus contained half the number of cells as the female transgenic thymus. The deletion of autospecific precursor cells in the male transgenic mouse began only at day 18 of gestation, despite the fact that the ligand could already be detected by day 16.

The preferential accumulation of CD4^-^8^+^ T cells, which expressed a high density of the transgenic TCR, occurred only after birth and was .obvious in 6-week-old female thymus. These data support the hypothesis that the positive selection of T cells expressing this transgenic heterodimer may involve two steps, i.e., the commitment of CD4^+^8^+^ thymocytes to the CD4^-^8^+^ lineage following the interaction of the transgenic TCR with restricting major histocompatibility molecules, followed by a slow conversion of CD4^+^8^+^ thymocytes into CD4^-^8^+^ T cells.

In normal mice, the precursors of CD^+^4^+^8 and single positive thymocytes have the CD4^-^8^-^ CD3^-^J11d^+^ (or M1/69 ^+^) phenotype. Because of the early expression of the transgenic αβ heterodimer, this population was not detected in adult transgenic mice. All CD4^-^8^-^ M1/ 69^+^ cells expressed the transgenic receptor associated with CD3 and could be readily grown in media containing T-cell lectins and interleukin 2.


*Developmental Immunology*, 1990, Vol. 1, pp. 1–10
Reprints available directly from the publisher
Photocopying permitted by license only

© 1990 Harwood Academic Publishers GmbH
Printed in the United Kingdom

### Early Deletion and Late Positive Selection of T Cells Expressing a Male-Specific Receptor in T-Cell Receptor Transgenic Mice

HUNG SIA TEH,*† HIROYUKI KISHI,‡ BERNADETTE SCOTT,‡ PETER BORGULYA,‡ HARALD VON BOEHMER‡ and PAWEL KISIELOW§

†*Department of Microbiology, University of British Columbia, Vancouver, Canada*
‡*The Basel Institute for Immunology, Basel, Switzerland*
§*The Institute for Immunology and Experimental Therapy, Polish Academy of Sciences, Wroclaw, Poland*

The ontogeny of T cells in T-cell receptor (TCR) transgenic mice, which express a transgenic $\alpha\beta$ heterodimer, specific for the male (H-Y) antigen in association with H-2D$^b$, was determined. The transgenic $\alpha$ chain was expressed on about 10% of the fetal thymocytes on day 14 of gestation. About 50% of day-15 fetal thymocytes expressed both $\alpha$ and $\beta$ transchains and virtually all fetal thymocytes expressed the transgenic $\alpha\beta$ heterodimer by day 17. The early expression of the transgenic TCR on CD4$^-$8$^-$ thymocytes prevented the development of $\gamma\delta$ cells, and led to accelerated growth of thymocytes and an earlier expression of CD4 and CD8 molecules. Up to day 17, no significant differences in T-cell development could be detected between female and male thymuses. By day 18 of gestation, the male transgenic thymus contained more CD4$^-$8$^-$ thymocytes than the female transgenic thymus. The preponderance of CD4$^-$8$^-$ thymocytes in the male transgenic thymus increased until birth and was a consequence of the deletion of the CD4$^+$8$^+$ thymocytes and their CD4$^-$8$^+$ precursors. By the time of birth, the male transgenic thymus contained half the number of cells as the female transgenic thymus. The deletion of autospecific precursor cells in the male transgenic mouse began only at day 18 of gestation, despite the fact that the ligand could already be detected by day 16.

The preferential accumulation of CD4$^-$8$^+$ T cells, which expressed a high density of the transgenic TCR, occurred only after birth and was obvious in 6-week-old female thymus. These data support the hypothesis that the positive selection of T cells expressing this transgenic heterodimer may involve two steps, i.e., the commitment of CD4$^+$8$^+$ thymocytes to the CD4$^-$8$^+$ lineage following the interaction of the transgenic TCR with restricting major histocompatibility molecules, followed by a slow conversion of CD4$^+$8$^+$ thymocytes into CD4$^-$8$^+$ T cells.

In normal mice, the precursors of CD4$^+$8$^+$ and single positive thymocytes have the CD4$^-$8$^-$ CD3$^-$J11d$^+$ (or M1/69$^+$) phenotype. Because of the early expression of the transgenic $\alpha\beta$ heterodimer, this population was not detected in adult transgenic mice. All CD4$^-$8$^-$ M1/69$^+$ cells expressed the transgenic receptor associated with CD3 and could be readily grown in media containing T-cell lectins and interleukin 2.

KEYWORDS: T cell development, T-cell receptor, transgenic mice, positive selection, negative selection.

### INTRODUCTION

During ontogeny, T-cell receptor (TCR) gene rearrangement and expression follow an ordered sequence. In mice, full-size transcripts of $\gamma$ and $\delta$ TCR genes are detected by day 14 of gestation (Brenner et al., 1988) and precede the appearance of full-size $\beta$ and $\alpha$ TCR gene transcripts that are formed by day 15 and day 17 of gestation, respectively (Raulet et al. 1985; Snodgrass et al., 1985a; Snodgrass et al., 1985b). TCR $\gamma\delta$ receptors are detected by day 15 (Brenner et al., 1988), and $\alpha\beta$ receptors by day 17 (Cristanti et. al., 1986).

*Corresponding author.

In $\beta$ TCR transgenic mice, we were unable to detect $\gamma\delta$ cells in the thymus. The TCR transgene prevented cell surface expression of all and rearrangement of certain $\gamma$ genes (V$\gamma$4/C$\gamma$4) (von Boehmer et al., 1988). This may be one of several reasons why usually $\gamma\delta$ and $\alpha\beta$ TCRs are not expressed in the same cell. Other evidence supports the view that the lineages of $\gamma\delta$ and $\alpha\beta$ TCR-expressing cells are determined prior to rearrangement of the $\delta$ locus: analysis of $\delta$-chain genes in $\alpha\beta$ T cells showed that the $\delta$ TCR locus exists in germline configuration in DNA circles that are excised during $\alpha$ TCR-gene rearrangement (Winoto and Baltimore, 1989). Because in our studies of $\beta$ TCR transgenic mice, all $CD4^-8^-$ thymocytes express the $\beta$ transgene (von Boehmer et al., 1988), we conclude that the expression of a rearranged $\beta$ TCR gene in cells of the $\gamma\delta$ lineage either prevents cell surface expression of $\gamma\delta$ genes or aborts further differentiation and expansion of this lineage. When we introduced rearranged TCR $\alpha$ and $\beta$ transgenes (Kisielow et al., 1988a), the same phenomenon was observed: $\gamma\delta$ cells were not detectable in these mice. In this report, we investigate the ontogeny of TCR-gene expression in the $\alpha\beta$ TCR transgenic mice in order to study whether an abnormally early expression of the $\alpha$ and $\beta$ TCR genes interfered with the development of the $\gamma\delta$ lineage.

The transgenic TCR was specific for the HY antigen in the context of H-$2D^b$ MHC molecules. In female TCR transgenic mice of the H-$2^b$ haplotype, T cells expressing the male-specific $\alpha\beta$ TCR were positively selected by the restricting H-$2D^b$ molecule in the absence of the male antigen and were of the $CD4^-8^+$ phenotype (Kisielow et al., 1988b; Teh et al., 1988). In male TCR transgenic mice of the H-$2^b$ haplotype, $CD4^+8^+$ immature thymocytes expressing the transgenic TCR were deleted (Kisielow et al., 1988a; Teh et al., 1989). Here we describe the sequence of phenotypic changes associated with positive and negative selection as they occur during ontogeny. Our conclusion from these studies is that negative selection can occur at the same stage or even on an earlier stage of thymocyte development as positive selection.

### RESULTS

### The T3.70 Antibody Binds to the Transgenic $\alpha$ TCR Chain in the Absence of the Transgenic $\beta$ Chain

In previous experiments, we could not really distinguish whether the T3.70 antibody binds to an idiotype defined by both the $\alpha$ and $\beta$ TCR chain or only the $\alpha$ TCR chain of the B6.2.16 clone from which the $\alpha$ and $\beta$ TCR genes were isolated (Uematsu et al., 1988). This was now determined by transfecting the $\alpha$ and $\beta$ TCR genes into the $CD4^+8^+$ thymona 110, which expresses its own $\alpha\beta$ TCR but which does not express $\beta$ TCR chains staining with the F23.1 monoclonal antibody and which does not stain with the T3.70 antibody. It turned out that some of the transfected cells expressed the transfected $\alpha$ TCR gene without concommitant expression of the transfected $\beta$ TCR gene. As these cells stained with the T3.70 antibody but not the F23.1 antibody, we conclude that the T3.70 antibody defines a determinant present on the transgenic $\alpha$ chain only (Fig. 1).

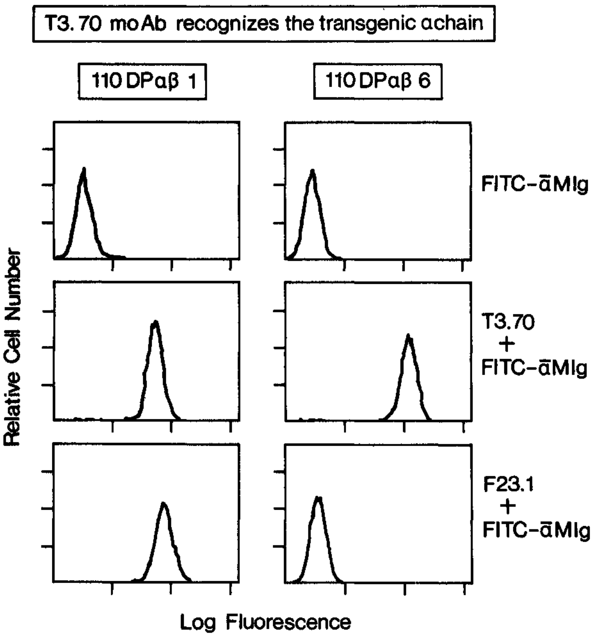

FIGURE 1. The T3.70 antibody stains T cells expressing the transgenic $\alpha$ but not the transgenic $\beta$ chain. The $CD4^+8^+$ (double-positive) thymoma 110 (110DP) was transfected with genes encoding the transgenic $\alpha$ and transgenic $\beta$ TCR chains. The transfectant 110DP$\alpha\beta$1 expressed both genes, whereas the transfectant 110DP$\alpha\beta$6 expressed the $\alpha$ genes only.

### Expression of $\beta$ and $\alpha$ TCR Transgenes by Embryonic Thymocytes

H-$2^b$ male $\alpha\beta$ TCR transgenic mice were mated with C57L (H-$2^b$) female mice and fetal thymuses from the embryos were collected at various times of gestation and analyzed for cell surface expression of the $\beta$ and $\alpha$ transgenic TCR chains by using the F23.1 and T3.70 antibodies respectively. It was verified in all experiments that thymocytes from nontransgenic C57L mice did not stain with either of the two monoclonal antibodies. As shown in Fig. 2, about 10% of thymocytes from the transgenic embryos were stained with the T.30 antibody. The proportion of thymocytes staining with both antibodies increased to 50% by day 15 of gestation. At day 19, it was noticed that practically all thymocytes stained with the $\beta$ TCR antibody and a fraction of these thymocytes did not stain with the $\alpha$ TCR antibody. This difference in staining by the two antibodies was also clearly evident at birth. Differences in the staining intensity of female and male mice were apparent by day 18 of gestation when the proportion of thymocytes expressing higher levels of the transgenic $\beta$ chain was increased in male embryos. Clearly, at birth, thymocytes expressing low levels of either $\beta$ or $\alpha$ TCR chains were absent from male mice.

The early expression of the $\alpha\beta$ heterodimer in transgenic mice also led to an accelerated growth of thymocytes. Between day 15 to 18 of gestation, the thymuses from TCR transgenic embryos contained twice the number of lymphocytes compared to nontransgenic littermates. There was no difference in the number of thymocytes recovered from female or male TCR transgenic thymuses up to day 17 of gestation. By the time of birth, however, the number of thymocytes recovered from male thymuses was less than half of that found in female thymuses, reflecting the deletion of $CD4^{+}8^{+}$ thymocytes and their $CD4^{-}8^{+}$ precursors (von Boehmer et al., 1988) in male offspring. By six weeks, only 10% of the number of thymocytes present in female mice was recovered from male TCR transgenic mice.

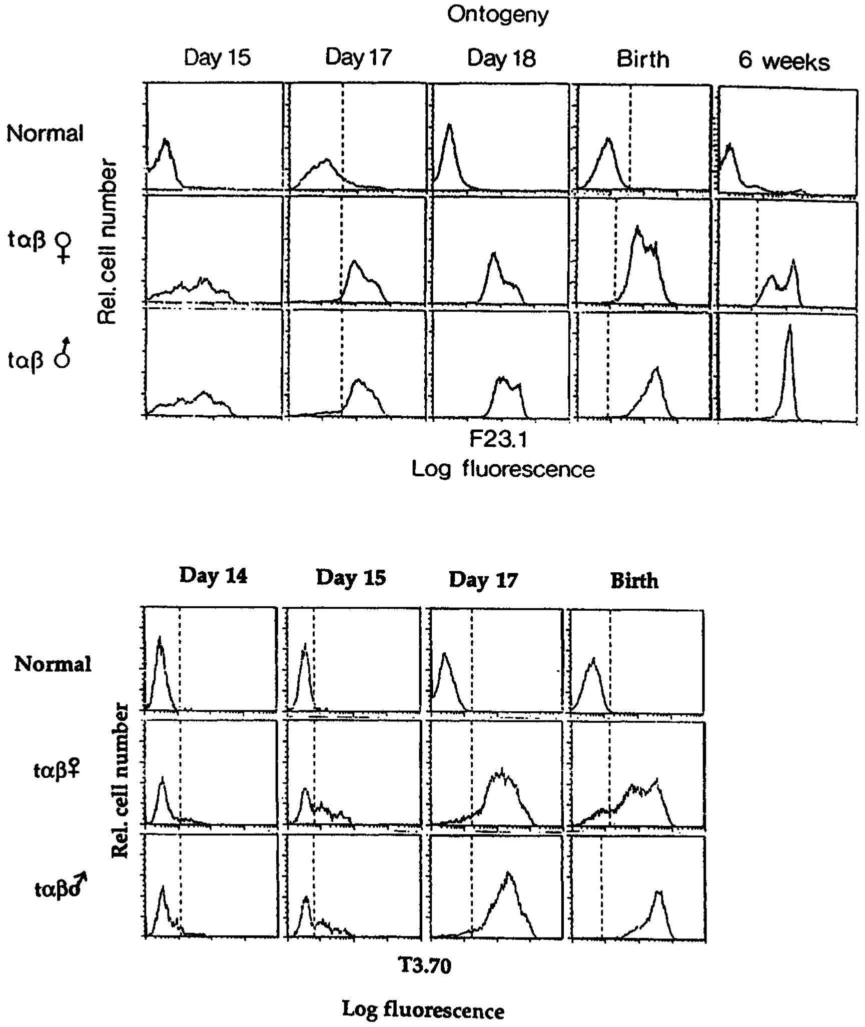

FIGURE 2. Ontogeny of expression of the $\beta$ and $\alpha$ transgenes. Female mice were mated with male $\alpha\beta$ transgenic mice, and fetal thymocytes arising from such matings were analyzed for surface expression of the transgenic $\beta$ or $\alpha$ chains at various times of gestation. The $\beta$ chain was stained by incubation with the F23.1 mAb followed by a second incubation with fluorescein-labeled sheep (Fab')2 fragment of antimouse Ig (Silenus Laboratory, Hawthorn, Australia). The transgenic $\alpha$ chain was stained by biotinylated T3.70 mAb followed by a second incubation with streptavidin phycoerythrin (Southern Biotechnology Associates, Birmingham, Alabama). The sex of the fetuses was determined by Southern blotting of fetal DNA with a male-specific probe (Lamar and Palmer, 1984). Thymocytes from 6-week-old female C57BL/6 mice were used as a nontransgenic (normal) control.

In summary, the $\beta$ and $\alpha$ TCR transgenes are coexpressed abnormally early in ontogeny and cause accelerated growth of embryonic thymocytes. After day 18, the thymus from male embryos contains less lymphocytes compared to the female thymus because of deletion of cells expressing lower levels of receptors.

### Ontogeny of M1/69 Positive and M1/69 Negative $CD4^-8^-$ Thymocytes in $\alpha\beta$ TCR Transgenic Mice

In the embryonic thymuses, all $CD4^-8^-$ thymocytes were M1/69 positive. The monoclonal M1/69 antibody detects the same molecular species as the J11d antibody. Thus, these early $CD4^-8^-$ thymocytes resemble the $CD4^-8^-$ precursor T cells in normal mice, which can give rise to all other thymocyte subpopulations. The only difference is that these cells in $\alpha\beta$ TCR transgenic mice express already high levels of the $\alpha\beta$ TCR. Until birth, we were unable to detect M1/69 negative $CD4^-8^-$ thymocytes in TCR transgenic mice, but by 6 weeks of postnatal life, a sizeable fraction (20%) of $CD4^-8^-$ cells were M1/69 negative but expressed the transgenic $\alpha\beta$ TCR. Thus, as in normal mice, this subset appeared relatively late in life despite the fact that $\alpha$ and $\beta$ TCR genes were already expressed very early in ontogeny (Fig. 3).

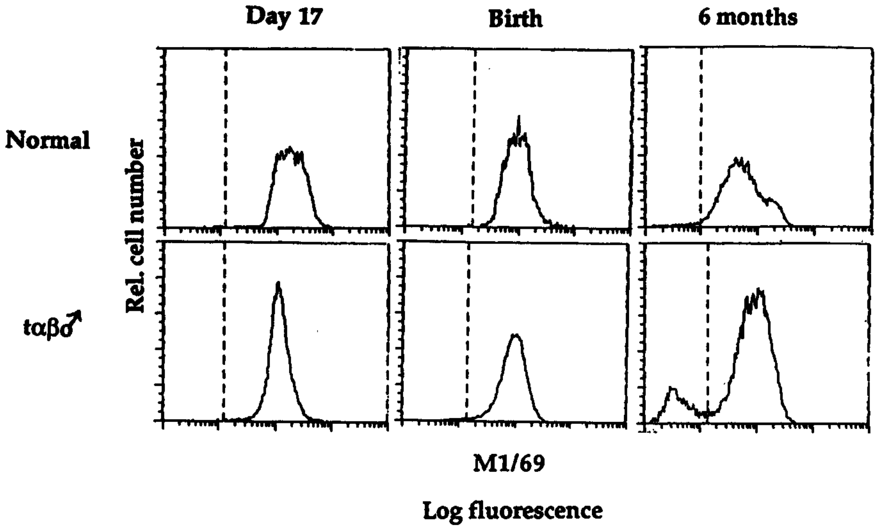

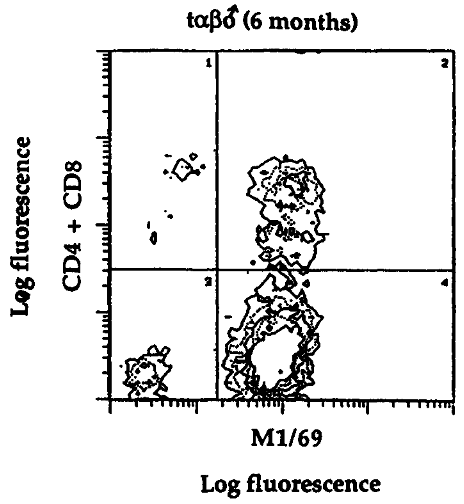

FIGURE 3. Late appearance of M1/69 negative thymocytes in male transgenic mice. Thymocytes of different ages from nontransgenic or transgenic male mice were stained with the M1/69 mAb and fluorescein-labeled sheep (FAB')2 fragment of antimouse Ig (top panel). In addition, 6-month-old male transgenic thymocytes were also stained with phycoerythrin-labeled CD4 and CD8 (Y axis) and with fluorescein-labeled M1.69 (X axis) (bottom panel). Contour plots were produced using the CONSORT-30 program from Becton-Dickinson.

### Both M1/69 Positive and M1/69 Negative $CD4^-8^-$ Thymocytes from TCR Transgenic Mice Can Be Induced to Proliferate

When cultured with either Concanavalin A, CD3, or TCR antibodies, as well as interleukin 2, both M1/69 positive and negative $CD4^-8^-$ thymocytes could be induced to proliferate. This means that the TCR/CD3 complex in the early M1/69 positive precursor T cells can transduce signals that lead to T-cell activation and proliferation (Table 1). This is in contrast to the in vivo $CD4^+8^+$ progeny of these cells, which cannot be induced to proliferate. This could mean that the signal transduction is changed when $CD4^-8^-$ cells differentiate into $CD4^+8^+$ thymocytes.

TABLE 1
Proliferation of $CD4^-8^-$, $M1/69^+$ and $M1/69^-$ Thymocytes from $\alpha\beta$ Transgenic Mice Induced by Concanavalin A or CD3 Antibodies[a]

| Responder cells ($1 \times 10^4$) | Concanavalin A (2.5 μg/ml) | CD3 antibodies (1 mg/ml) | — |
|---|---|---|---|
| $CD4^-8^-$, $M1/69^+$ | 15,400 | 8.300 | 0.300 |
| $CD4^-8^-$, $M1/69^-$ | 35,700 | 16.600 | 1.100 |

[a]All cultures contained $10^6$ X-irradiated C57B1/6 nu/nu spleen cells and interleuken 2.

### T-Cell Development in $\alpha\beta$ TCR Transgenic Mice

Thymocytes from normal mice can be subdivided into four categories on the basis of staining with CD4 and CD8 antibodies, namely, $CD4^-8^-$, $CD4^+8^+$, $CD4^+8^-$, and $CD4^-8^+$ thymocytes. Mature T cells with $\alpha\beta$ TCRs are either $CD4^+8^-$ or $CD4^-8^+$. $CD4^-8^-$ thymocytes contain the precursors for all other subsets (Kisielow et al., 1984; Fowlkes et al., 1985), whereas $CD4^+8^+$ thymocytes contain the precursors of $CD4^+8^-$ and $CD4^-8^+$ thymocytes (Kisielow et al., 1988a; Nicolic-Zugic and Bevan, 1988). The double staining patterns of female and male transgenic thymocytes as well as thymocytes at various times of gestation with CD4 and CD8 mAb are shown in Fig. 4. By day 15 of gestation, most cells were double negative, but there was a higher proportion of cells expressing CD4 and CD8 co-receptors in thymuses from the transgenic mice. By day 17, the vast majority of thymocytes were $CD4^+8^+$ and there was no significant difference between the thymus from female and male transgenic mice. By day 18, the proportion of double-negative thymocytes in the male thymus was noticeably larger than in female transgenic littermates. The difference between female and male transgenic

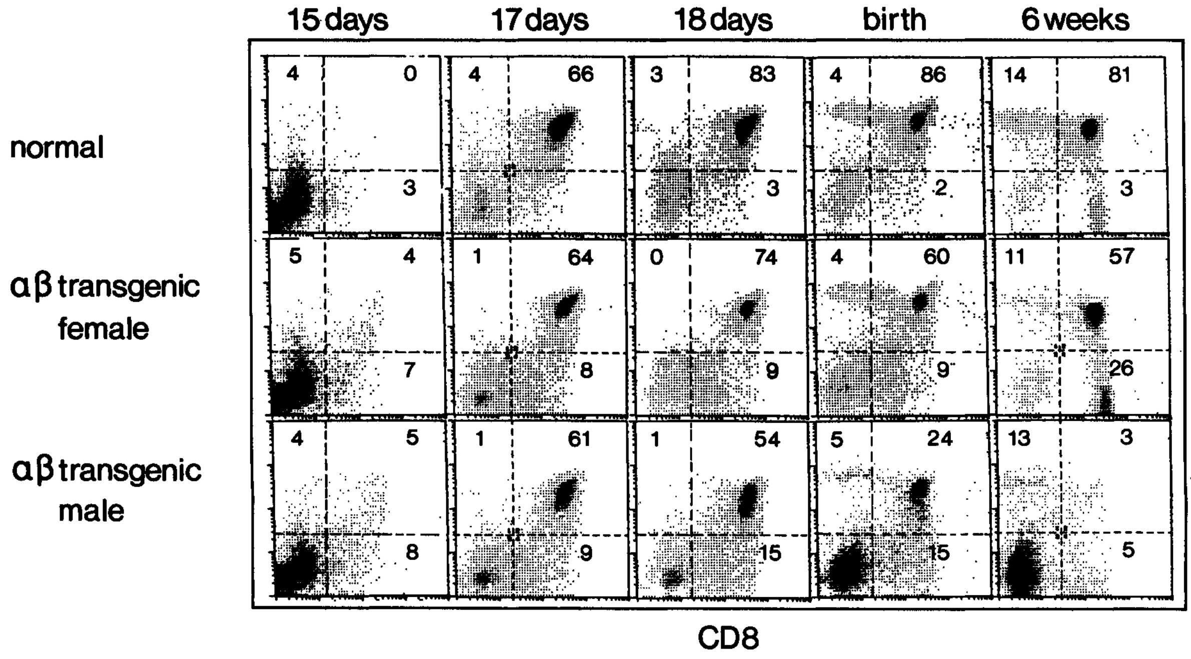

FIGURE 4. Ontogeny of T cells in nontransgenic and transgenic mice. Thymocytes of the indicated age were stained with phycoerythrin-labeled CD4 and fluorescein-labeled CD8 mAb and analyzed in the FACScan flow cytometer, as previously described (Kisielow et al., 1988a; Teh et al., 1988).

thymuses was especially pronounced in newborn animals, where the majority of male thymocytes were CD4⁻8⁻. Although the signs of deletion were clearly apparent at birth, the phenotypic changes associated with positive selection were not yet evident. All thymuses contained a significant population of $CD4^{+}8^{-}$ thymocytes at this time, but $CD4^{-}8^{+}$ thymocytes were not yet present in significant numbers even in the thymus from female transgenic newborns. The overrepresentation of $CD4^{-}8^{+}$ thymocytes, as noticed previously, was, however, clearly apparent 6 weeks after birth.

The expression of $\alpha$ and $\beta$ transgenic TCR chains and CD4 and CD8 coreceptors are shown in Fig. 5. By day 17, the female and male thymuses from transgenic embryos contained two major populations, namely, $CD4^{-}8^{-}$ $\alpha T^{hi}\beta T^{hi}$ and $CD4^{+}8^{+}$ $\alpha T^{lo}\beta T^{lo}$. At birth, the female transgenic thymus contained, in addition to the above populations, a discrete population of $CD4^{+}8^{-}$ cells that expressed high levels of the transgenic $\beta$ chain, but lacked the transgenic $\alpha$ chain. This population was lacking in the male transgenic thymus, which contained only cells expressing high levels of the receptor, some of which stained only faintly with CD4 or CD8 antibodies.

In summary, these data indicate that the phenotypic changes associated with deletion (deletion of $CD4^{+}8^{+}$ thymocytes) are apparent in embryonic thymuses from day 18 onwards, whereas the pheno-

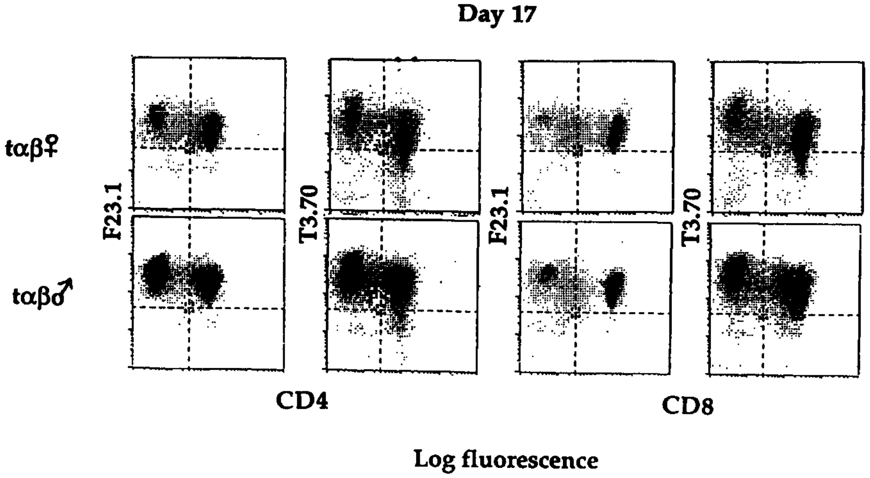

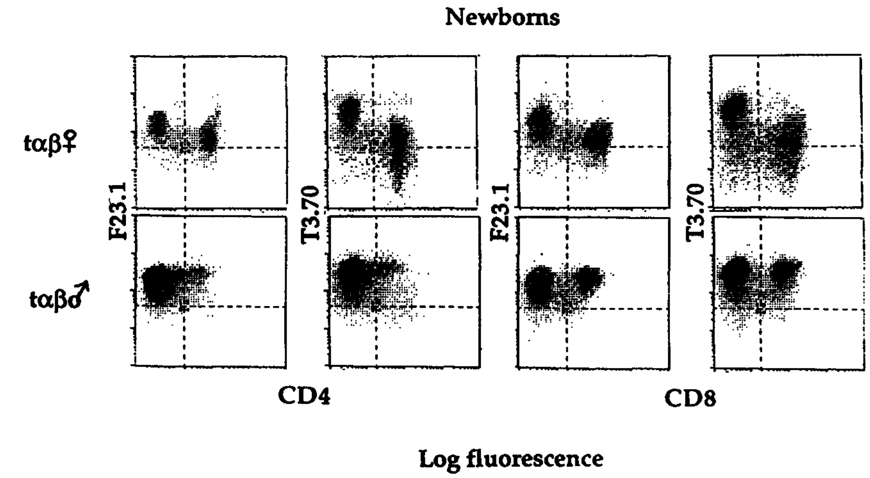

FIGURE 5. Deletion of autospecific precursors in male transgenic neonates. Thymocytes from day-17 fetuses or neonates were double stained either with the CD4 mAb and F23.1 or T3.70 mAb or with the CD8 mAb and F23.1 or T3.70 mAb. This was achieved by incubating the thymocytes first with fluorescein-labeled CD4 or CD8 followed by a second incubation with biotinylated F23.1 or T3.70 mAb and a third incubation with streptavidin phycoerythrin.

typic changes associated with positive selection (an increased proportion of $CD4^-8^+$ thymocytes) are only visible well after birth.

### Ontogeny of the Ligand for the Transgenic TCR and Onset of Deletion

Because we did not find any sign of deletion of $CD4^+8^+$ thymocytes by day 17 of gestation, we wondered if this was due to a late appearance of the ligand in the embryonic thymus. This was tested by culturing the B6.2.1 clone from which the $\alpha$ and $\beta$ transgenes were obtained with X-irradiated thymocytes from 16-day-old embryos and interleukin 2. The individual embryos were sexed using a male-specific probe (Lamar and Palmer, 1984). As shown in Table 2, the ligand was expressed on thymocytes from 16-day-old embryos as the thymocytes from male embryos induced significant proliferation in the B6.1.16 clone. Thus, the ligand was present 2 days earlier before we could detect the first signs of deletion, which resulted in a decreased number of $CD4^+8^+$ thymocytes.

TABLE 2
Stimulation of the Male-Specific B6.2.16 Clone by Spleen Cells and Thymocytes from Day-16 Embryos

| Female Stimulators | cpm[a] | Male Stimulators | cpm |
|---|---|---|---|
| $1 \times 10^5$ spleen | 0.800 | $1 \times 10^5$ spleen | 7.200 |
| $<1 \times 10^5$ embryonic thymocytes 1/2 | 1.000 | $<1 \times 10^5$ embryonic thymocytes 1/1 | 3.700 |
| 1/4 | 0.700 | 1/3 | 3.200 |
| 1/7 | 0.900 | 1/5 | 3.300 |
| 2/1 | 0.600 | 1/6 | 5.100 |
| 2/2 | 0.600 | 1/8 | 2.400 |
| 2/4 | 1.000 | 2/3 | 4.600 |
| | | 2/5 | 4.400 |

[a]In addition to stimulators, all cultures contained $1 \times 10^4$ B6.2.16 cells and IL2.

### DISCUSSION

The data on the ontogeny of the expression of the transgenic TCR indicate that both the $\beta$ and the $\alpha$ TCR chain expressed relatively early on $CD4^-8^-$ M1/$69^+$ thymocytes by day 14 to 15 of gestation. One reason for this is that already rearranged genes are expressed earlier than rearranging genes. In fact, it was noted by some investigators that even in normal mice, some "early" $CD4^-8^+$ precursors of $CD4^+8^+$ thymocytes express CD3 molecules presumably associated with the $\alpha\beta$ TCR (Nicolic-Zugic et al., 1988). Another reason for the early expression of the TCR $\alpha$ chain might be that its expression is regulated by the $\beta$ TCR enhancer located 5 kb downstream of $C\beta_2$: it is conceivable that the random integration of both the $\beta$ TCR and $\alpha$ TCR DNA into the genome of the transgenic mice has occurred in such a way that the expression of both genes is regulated by the same $\beta$ TCR enhancer. This view is supported by observations made in mice expressing the $\alpha$ transgene only: here much fewer cells express the transgene and this is the case even when $\alpha$ and $\beta$ transgenic mice are crossed to exclude preferential pairing as reasons for the high expression of the transgenic $\alpha$ TCR chain in $\alpha\beta$ transgenic mice. Thus, two reasons: first, the fact that the $\alpha$ TCR gene is already rearranged, and, second, expression, which is enhanced by the $\beta$ TCR enhancer, may contribute to the early expression of the $\alpha\beta$ TCR enhancer, may contribute to the early expression of the $\alpha\beta$ TCR on the majority of $CD4^-8^-$ M1/$69^+$ thymocytes from day 17 onwards. Obviously, most of the $CD4^-8^-$ M1/$69^+$ cells can express the CD3 complex that is associated with the $\alpha\beta$ TCR. It appears that a significant proportion of the $CD4^-8^-$ M1/$69^+$ cells expressing the $\alpha\beta$ TCR can be induced by antibodies against the TCR complex to express lymphokine receptors and proliferate in lymphokine-containing media. This is, however, not so with $CD4^+8^+$ thymocytes expressing the $\alpha\beta$ TCR associated with CD3. This could have two possible interpretations: one is that the TCR-signal transduction is altered when $CD4^+8^+$ thymocytes are formed from $CD4^-8^-$ precursors. The other is that only a subset of $CD4^-8^-$ J11d$^+$ thymocytes can be induced to proliferate. One could, for instance, speculate that these are cells of the $\gamma\delta$ lineage that express the $\alpha\beta$ TCR transgenes. This needs to be studied in more detail.

It is clear that the early expression of the $\alpha\beta$ TCR prevents the development of the $\gamma\delta$ lineage. Because in mice markers other than the TCR cannot be used to identify the $\gamma\delta$ lineage, it is not certain whether this is due to an abortive differentiation of this lineage or to a block of expression and/or rearrangement of the $\gamma\delta$ TCR in that lineage.

The early expression of the $\alpha\beta$ TCR leads to some acceleration in T-cell development, i.e., the earlier expression of CD8 molecule and an accelerated growth. This is well in line with observations in *scid* mice into which rearranged TCR genes were introduced by breeding and that resulted in the expression of CD8 and CD4 coreceptors as well as an enormous expansion of thymocytes in vivo (Scott et al., 1989).

The analysis of the thymus at various stages of gestation clearly indicates that the phenotypic changes associated with negative selection are apparent much earlier than those associated with positive selection. Although there are no significant differences between the male and female thymuses up to day 18, the deletion of $CD4^{+}8^{+}$ thymocytes and their $CD4^{-}8^{+}$ precursors became apparent at that time such that at birth the thymus contained mostly $CD4^{-}8^{-}$ thymocytes and a much reduced lymphocyte population. Despite the fact that the ligand for the transgenic TCR could be detected already by day 16 of gestation, we were unable to detect deletion before day 18. This could mean that the deletion requires a certain antigen-presenting cell that develops relatively late during ontogeny or that the cells themselves develop the mechanism leading to deletion relatively late. We prefer the former possibility because in adult life even the earliest stages of newly developing $CD4^{+}8^{+}$ thymocytes are deleted, i.e., these cells are equipped with the program that leads to cell death.

In contrast to negative selection, the phenotypic changes associated with positive selection develop quite late in that the preponderance of $CD4^{-}8^{+}$ thymocytes is only apparent well after birth. Although these findings are consistent with the view that negative selection can precede positive selection during T-cell development, they do not provide firm evidence that this is the case. The transgenic mice, however, provide evidence that negative selection can occur at the same or earlier stages of T-cell development than positive selection: male transgenic mice lack or have much more reduced numbers of $CD4^{+}8^{-}$ thymocytes that are *not* male-specific and express endogenous $\alpha$ TCR chains (Kisielow et al., 1988a; Teh et al., 1989; and Fig. 5). These cells are obviously absent in male mice because their precursors, namely, $CD4^{+}8^{+}$ thymocytes, still expressing the transgenic TCR, have been deleted. This is well in line with out observation that $CD4^{+}8^{-}$ cells in female $\alpha\beta$ transgenic mice express transgenic as well as endogenous $\alpha$ TCR transcripts (findings to be published). Thus, negative selection can delete precursors of cells that would be positively selected, indicating that susceptibility to negative selection can occur at the same developmental stage as susceptibility to positive selection.

### MATERIALS AND METHODS

### Mice

C57BL/6 (H-$2^{b}$) and C57L (H-$2^{b}$) mice were purchased from the Jackson Laboratory, Bar Harbor, Maine. TCR transgenic mice expressing both the $\alpha$ and $\beta$ genes, which were isolated from a cytolytic T-cell clone with specificity for the male antigen in association with H-$2D^{b}$, were produced as previously described (Kisielow et al., 1988a; Uematsu et al., 1988). The $\alpha\beta$ transgenic mice used in the studies reported here had been backcrossed for five to six generations to C57L female mice.

### Ontogeny Studies

Male $\alpha\beta$ transgenic mice were mated with C57L females. The day of which a vaginal plug was observed was designated as day 0. At various times of gestation, fetal thymuses were collected and analyzed individually for expression of specific cell surface molecules. Single cell suspensions of both fetal thymus lobes from individual animals were prepared by rubbing the thymuses between two pieces of 1 $cm^2$ nylon with 40-$\mu$ mesh size. Transgenic and nontransgenic fetuses were distinguished on the basis of expression of the transgenic $\alpha$ and $\beta$ chains on thymocytes.

### Monoclonal Antibodies (mAb)

Phycoerythrin-labeled CD4 (antimouse L3T4) (Dialynas et al., 1983), biotinylated CD8 (antimouse Lyt-2) (Ledbetter and Herzenberg, 1979), and fluorescein-labeled CD8 mAbs were purchased from Becton Dickinson, Mountain View, California. Fluorescein-labeled CD4 was a kind gift of Dr. Jeffrey Ledbetter, Oncogen, Seattle. The F23.1 (Staerz et al., 1985) and T3.70 (Teh et al., 1989) mAbs were purified by protein A chromatography and used either in the unconjugated or biotinylated form. The M1/69 hybridoma cells (Springer et al., 1978) were obtained from Dr. Fumio Takei, Terry Fox Laboratory, Vancouver. Spent culture supernatant from this hybridoma line was used for staining of thymocytes.

### Staining of Thymocytes

Single or two-color staining of thymocytes was performed as previously described. The stained thymocytes were analyzed with a FACScan Flow

Cytometer (Becton Dickinson) as previously described (Kisielow et al., 1988a; Teh et al., 1988).

### Transfection of $\alpha$ and $\beta$ TCR Genes Into T-Cell Lines

Ten $\mu$g each of cosmid DNA that contained the functional TCR $\alpha$ chain gene (COSHY$\alpha$36) or the TCR$\beta$ chain gene (COSHY$\beta$9.1.14) was linearized by PvuI digestion. After digestion, DNAs were dissolved in 400 $\mu$l of PBS and added to 400 $\mu$l of $2 \times 10^6$ 110TC $CD4^+8^+$ thymoma in PBS. COSHY$\alpha$36 and COSHY$\beta$9.1.14 were cotransfected by electroporation using a Gene Pulser (Bio-Rad) under the condition of 960 $\mu$F and 250 V. After transfection, cells were cultured in medium for 2 days, and then transformants were selected in media containing 2 mg/ml G418.

### Isolation of $CC4^-CD8^-$ Thymocytes

$CD4^-CD8^-$ cells were isolated by treating cells (at a concentration of $10^7$ cells/ml in RPMI medium plus 5% FCS) with previously determined optimal concentrations of the anti-CD8 monoclonal antibody HO2.2 (Gottlieb et al., 1980) and the anti-CD4 monoclonal antibody RL172 (Ceredig et al., 1985) for 30 min, on ice. Rabbit complement (Cedar Lane Laboratories, Canada) was added to a final concentration of 1/10 and the cells were incubated for 45 min at 37°C. Remaining viable cells were subjected to a second depletion step to ensure the purity of the $CD4^-8^-$ population. This process involved incubating the cells ($5 \times 10^6$/ml in PBS plus 5% FCS) with the anti-CD8 monoclonal antibody 53.6.7 (Ledbetter and Herzenberg, 1979) and the anti-CD4 monoclonal antibody GK1.5 (Dialynas et al., 1983) for 30 min on ice. The cells were washed twice, resuspended to $5 \times 10^6$ cells/ml and incubated with a predetermined optimal concentration of Dynabeads (magnetic beads, Dynal, Norway) conjugated to sheep antimouse Ig. The cells and beads were rotated for 30 min at 4°C and then subjected to magnetic separation.

### Dot Blot Analysis

Embryos were homogenized in 0.5 M EDTA, 100 $\mu$g/ml Proteinase K, 0.5% Sarcosyl, and 100 $\mu$g/ml RNase A using a Polytron with a PTA7 blade. DNA was isolated by phenolchloroform extraction and dialyzed against 10 mM Tris, 1 mM EDTA (pH 8.0). Five $\mu$g DNA was ethanol precipitated, alkaline denatured at 80°C, and transferred to a nitrocellulose membrane (BA85, Schleicher & Schuell) using a Minifold microsample filtration manifold (Schleicher & Schuell). After baking for 2 hr at 65°C in 1.5 ×SSPE, 1% SDS, 0.5% BLOTTO, and 50-$\mu$g/ml salmon sperm, DNA hybridization to the P–Y probe (Lamar and Palmer, 1984) was performed overnight at 65°C using the prehybridization buffer, including additional 10% Dextrane Sulfate. The membrane was washed twice with 3 ×SSC, 0.1% SDS, and twice with 0.1 ×SSC, 0.1% SDS at 60°C prior to autoradiography.

### Culture of Embryonic Thymocytes

$5 \times 10^4$ thymocytes were cultured in 0.2-ml tissue-culture medium containing interleukin 2 and $2 \times 10^5$ X-irradiated spleen cells from nu/nu mice. Some of the cells contained conconavalin A (2 $\mu$g/ml), CD3 antibodies (Leo et al., 1987) or TCR antibodies (F23.1) at 1 $\mu$g/ml.

### ACKNOWLEDGMENTS

The expert technical assistance by Katrin Hafen and Verena Stauffer is gratefully acknowledged. We thank Dr. Horst Blüthmann for some timed matings and the sexing of some embryos. We thank Nicole Schoepflin for typing the manuscript. The Basel Institute for Immunology was founded and is supported by F. Hoffmann–La Roche Ltd., Basel, Switzerland.

*(Received October 15, 1989)*

*(Accepted October 23, 1989)*

### REFERENCES

Brenner M.B., Strominger J.L., and Krangel M.S. (1988). The $\gamma\delta$ T cell receptor. Adv. Immunol. **43:** 133.

Ceredig R., Lowenthal J.W., Nabholz M., and MacDonald H.R. (1985). Expression of interleukin-2 receptors as a differentiation marker on intrathymic stem cells. Nature **314:** 98.

Crisanti A., Colantoni A., Snodgrass R., and von Boehmer, H. (1986). Expression of T cell receptors: in situ staining and biochemical analysis. EMBO J. **5:** 2837.

Crispe I.N., and Bevan M.J. (1987). Expression and functional significance of the J11d marker on mouse thymocytes. J. Immunol. **138:** 2013.

Dialynas D.P., Wilde D.B., Marrack P., Pierres A., Wall K.A., Havran W., Otten G., Lokan M.R., Pierres M., Kappler J. and Fitch F.W. (1983). Characterization of the murine antigenic determinant, designated L3T4a, recognized by monoclonal antibody GK1.5: expression of L3T4a by functional T cell clones appears to correlate primarily with class II MHC antigen reactivity. Immunol. Rev. **74:** 29.

Fowlkes B.J., Edison L., Mathieson B.J. and Chused T.M. (1985). Early T lymphocytes. J. Exp. Med. **162:** 802.

Gottlieb P., Marshak-Rothstein A., Anditore-Hargreaves K., Berkoben D.B., August D.A., Rosche R.M., and Benedetto J.D. (1980). Construction and properties of new Lyt-congenic strains and anti-Lyt-2.2 and anti-Lyt-3.1 monoclonal antibodies. Immunogenetics **10:** 544.

Kisielow P., Blüthmann H., Staerz U.D., Steinmetz M., and von Boehmer H. (1988a). Tolerance in T-cell receptor transgenic mice involves deletion of nonmature $CD4^{+}8^{+}$ thymocytes. Nature **333:** 742.

Kisielow P., Teh H.S., Blüthman H., and von Boehmer H. (1988b). Positive selection of antigen-specific T cells in thymus by restricting MHC molecules. Nature **335:** 730.

Lamar E.G., and Palmer E. (1984). Y-encoded, species-specific DNA in mice: evidence that the Y chromosome exists in two polymorphic forms in inbred mice. Cell **47:** 171.

Ledbetter J.A., and Herzenberg L.A. (1979). Xenogeneic monoclonal antibodies to mouse lymphoid differentiation antigens. Immunol. Rev. **47:** 63.

Leo O., Foo M., Sachs D.H., Samelson L.E., and Bluestone J.A. (1987). Identification of a monoclonal antibody specific for a murine T3 polypeptide. Proc. Natl. Acad. Sci. USA **84:** 1374.

Nicolic-Zugic J., and Bevan M.J. (1988). Thymocytes expressing CD8 differentiate into $CD4^{+}$ cells following intrathymic injection. Proc. Natl. Acad. Sci. USA **85:** 8633.

Raylet D.H., Gorman R.D., Saito H., and Tonegawa S. (1985). Developmental regulation of T-cell receptor gene expression. Nature **314:** 103.

Scott B., Blüthman H., Teh H.S., and von Boehmer H. (1989). The generation of mature T cells requires interaction of the $\alpha\beta$ T-cell receptor with major histocompatibility antigens. Nature **338:** 591.

Snodgrass H.R., Kisielow P., Kiefer M., Steimetz M., and von Boehmer H. (1985a). Ontogeny of the T-cell antigen-receptor within the thymus. Nature **313:** 592.

Snodgrass H.R., Dembic Z., Steinmetz M., and von Boehmer H. (1985b). Expression of T-cell antigen-receptor genes during fetal development in the thymus. Nature **315:** 332.

Springer T., Galfrie G., Secher D.S., and Milstein C. (1978). Monoclonal xenogeneic antibodies to murine cell surface antigens: identification of novel leukocyte differentiation antigens. Eur. J. Immunol. **8:** 539.

Staerz U., Rammensee H., Benedetto J., and Bevan M.J. (1985). Characterization of a murine monoclonal antibody specific for an allotype determinant on T cell antigen receptor. J. Immunol. **134:** 3994.

Teh H.S., Kisielow P., Scott B., Kishi H., Uematsu Y., Blüthman H., and von Boehmer H. (1988). Thymic major histocompatibility complex antigens and the $\alpha\beta$ T-cell receptor determine the CD4/CD8 phenotype of T cells. Nature **335:** 229.

Teh H.S., Kishi H., Scott B., and von Boehmer H. (1989). Deletion of autospecific T cells in T cell receptor transgenic mice spares cells with normal TCR levels and low levels of CD8 molecules. J. Exp. Med. **169:** 795.

Uematsu Y., Ryser S., Dembic Z., Borgulya P., Krimpenfort P., Berns A., von Boehmer H., and Steinmetz M. (1988) In transgenic mice the introduced functional T cell receptor $\beta$ chain prevents expression of endogenous $\beta$ genes. Cell **52:** 831.

von Boehmer H., Bonneville M., Ishida I., Ryser S., Lincoln G., Smith R.T., Kishi H., Scott B., Kisielow P., and Tonegawa S. (1988). Early expression of a T cell receptor $\beta$ chain transgene suppresses rearrangement of the $V\gamma4$ gene segment. Proc. Natl. Acad. Sci. USA **85:** 9729.

von Boehmer H. (1988) The developmental biology of T lymphocytes. Annu. Rev. Immunol. **6:** 309.

Winoto A., and Baltimore D. (1989). Separate lineages of T cells expressing the $\alpha\beta$ and $\gamma\delta$ receptors. Nature **338:** 430.

